# Invasive pressure–volume loop and PET-MR phenotyping in transthyretin cardiac amyloidosis: a multimodal imaging case report

**DOI:** 10.1093/ehjcr/ytag085

**Published:** 2026-02-04

**Authors:** Ashwin Venkateshvaran, Björn Pilebro, Sandra Arvidsson, Fredrik Edbom, Per Lindqvist

**Affiliations:** Department of Diagnostics and Intervention, Clinical Physiology, Umeå University, Norrlands Universitetssjukhus, 901 85 Umeå, Sweden; Department of Diagnostics and Intervention, Clinical Physiology, Umeå University, Norrlands Universitetssjukhus, 901 85 Umeå, Sweden; Department of Diagnostics and Intervention, Clinical Physiology, Umeå University, Norrlands Universitetssjukhus, 901 85 Umeå, Sweden; Department of Diagnostics and Intervention, Clinical Physiology, Umeå University, Norrlands Universitetssjukhus, 901 85 Umeå, Sweden; Department of Diagnostics and Intervention, Clinical Physiology, Umeå University, Norrlands Universitetssjukhus, 901 85 Umeå, Sweden

**Keywords:** Echocardiography, Cardiac MRI, DPD scintigraphy, Myocardial work index, Pressure–volume loop, Case report, Amyloidosis

## Abstract

**Background:**

Cardiac amyloidosis (CA) is an under-recognized cause of heart failure in elderly patients. While diagnosis has traditionally relied on echocardiographic red flags and bone scintigraphy, novel tools may provide enhanced disease characterization.

**Case summary:**

We present the case of a 78-year-old man with progressive symptoms of heart failure who was diagnosed with wild-type transthyretin cardiac amyloidosis (ATTRwt-CM) through conventional and advanced multimodal imaging. Initial clues included a discordance between QRS voltages on electrocardiography and increased left ventricular wall thickness on echocardiography, along with signs of elevated filling pressures. Speckle-tracking echocardiography revealed impaired regional myocardial deformation, global function, and work energetics. Serum and urine immunofixation excluded light chain (AL) amyloidosis. DPD scintigraphy confirmed amyloid deposition with a characteristic distribution. Genetic testing ruled out hereditary variants. PET imaging demonstrated myocardial uptake suggestive of amyloid infiltration and microcalcification. Cardiac MR revealed elevated native T1 and extracellular volume fractions. Invasive pressure–volume loop assessment confirmed biventricular stiffness and impaired contractile reserve, despite clinical compensation at rest. These findings supported early initiation of Tafamidis in a minimally symptomatic patient.

**Discussion:**

This case highlights the role of advanced diagnostics in refining cardiac amyloidosis phenotyping and guiding individualized therapeutic decisions.

Learning points

- Multimodal cardiac imaging can detect myocardial involvement in transthyretin amyloid cardiomyopathy (ATTRwt-CM) even before overt heart failure symptoms develop.
- Hybrid PET–MR provides complementary information on amyloid burden and microcalcification beyond echocardiography and DPD scintigraphy.
- Pressure–volume loop analysis quantifies myocardial stiffness and contractile reserve, offering a potential tool to guide early therapeutic intervention.

## Introduction

Transthyretin Cardiac Amyloidosis (ATTR-CM) is increasingly recognized but routinely underdiagnosed in heart failure. Advances in imaging permit accurate, non-invasive diagnosis of ATTR-CM, reducing reliance on confirmatory endomyocardial biopsy. Furthermore, integration of medical history with multimodal imaging is crucial for early detection, therapy selection, and monitoring treatment outcomes in an era of expensive, disease-modifying treatments.

Although clinical trials have demonstrated therapeutic efficacy in symptomatic patients,^1,2^ high treatment costs often restrict reimbursement to those who strictly meet selection criteria. A particularly challenging clinical scenario arises when patients are relatively asymptomatic despite imaging findings suggestive of advanced myocardial involvement. This discordance complicates clinical decision-making and emphasizes the critical role of imaging in providing objective evidence of disease severity and supporting timely intervention.

We present a case of wild-type ATTR-CM (ATTRwt-CM), in which emerging technologies such as PET-MR imaging and invasive biventricular PV-loop assessment provided complementary diagnostic insights and supported clinical decision making.

## Summary figure

**Figure:**
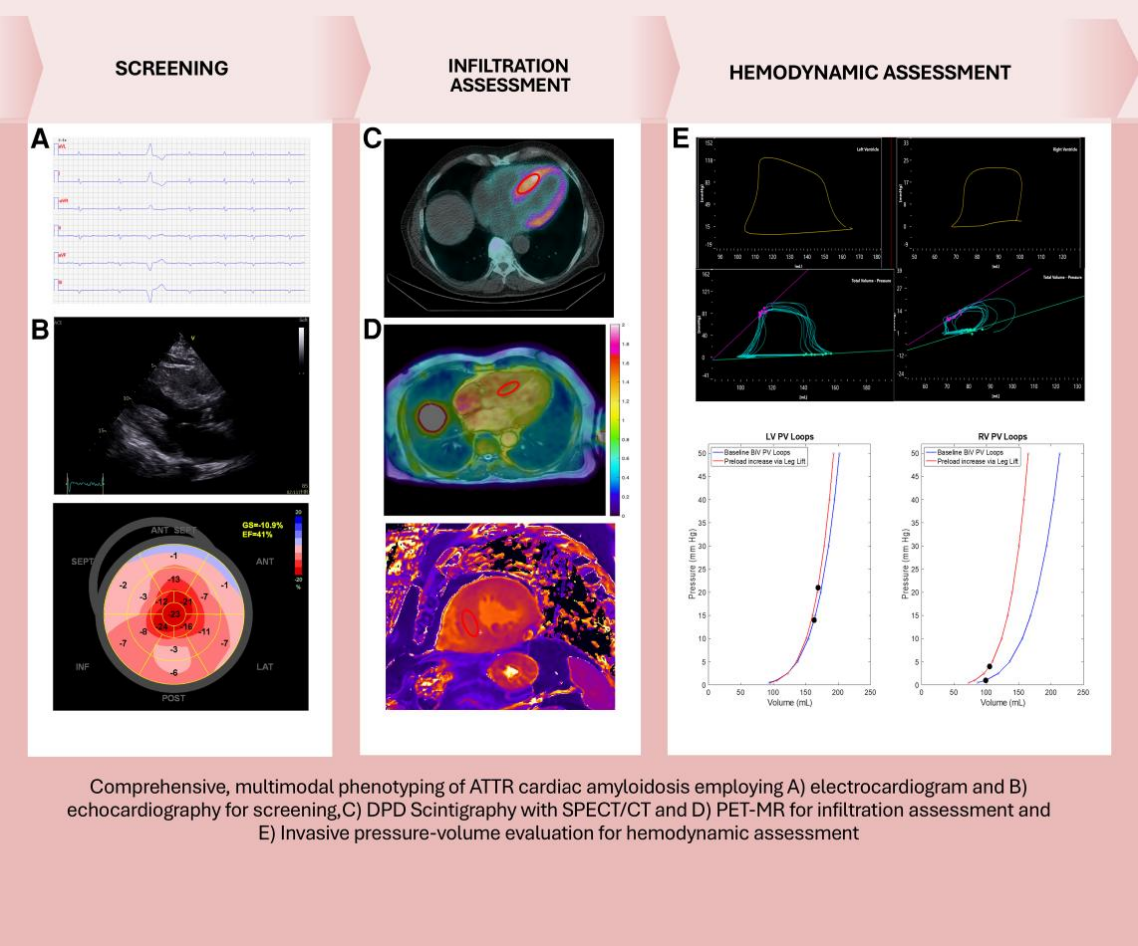


## Case presentation

A 78-year-old man (height 174 cm, weight 80 kg) presented in March 2024 with exertional dyspnoea and peripheral oedema coinciding with a viral respiratory tract infection. Following recovery from the infection, his heart failure symptoms spontaneously regressed without treatment. His medical history included well-controlled hypertension (blood pressure 125/75 mmHg on presentation). Cardiac auscultation was unremarkable, and heart rate was regular at 76 beats per minute.

**Figure 1:**
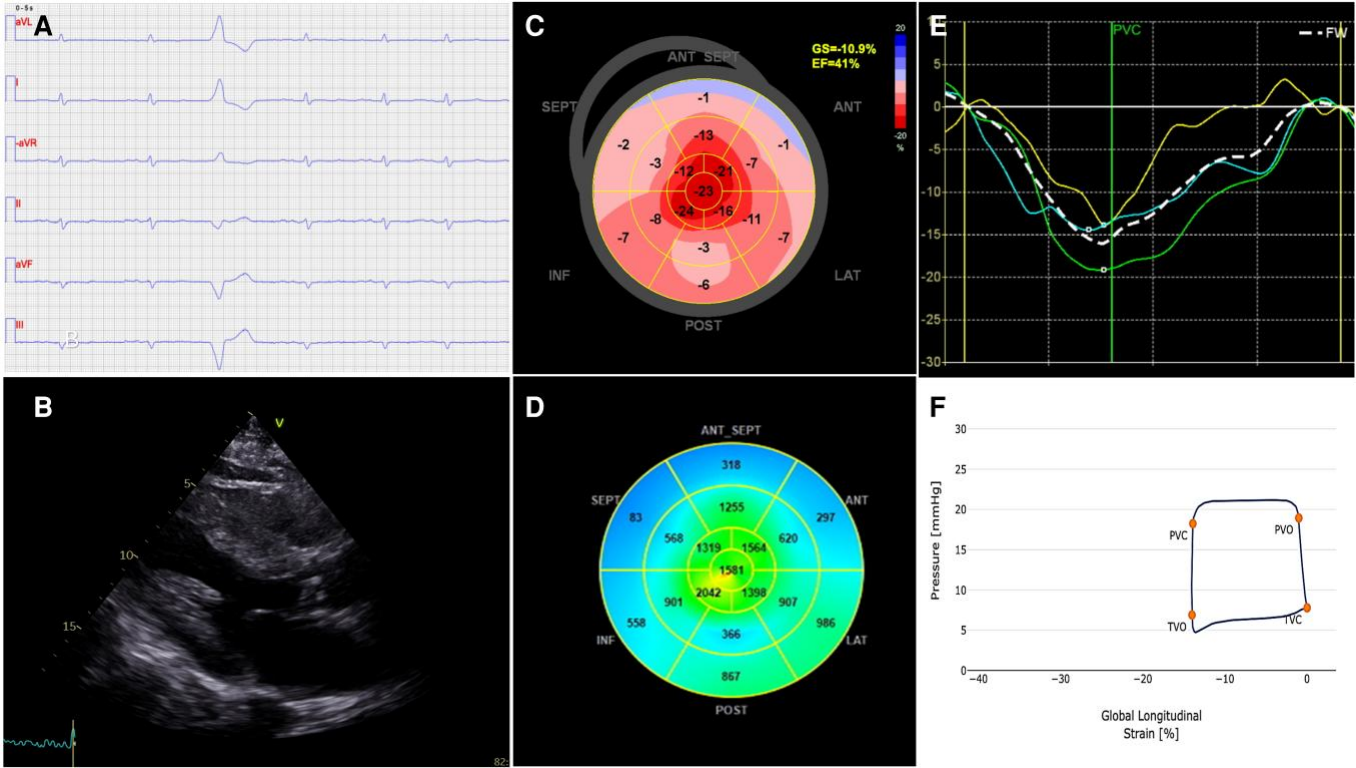
Electrocardiographic and transthoracic echocardiographic screening of ATTR cardiac amyloidosis. *A*) ECG displaying low QRS complexes, *B*) 2D echocardiography demonstrating left ventricular hypertrophy, *C*) Strain Imaging displaying a reduced GLS and apical sparing, *D*) Bulls Eye suggesting reduced myocardial work index, *E*) RV strain curves suggesting reduced free wall strain, *F*) RV pressure–strain loop in ATTR-CM.

In May 2024, the patient’s N-terminal pro-B-type natriuretic peptide (NT-proBNP) was elevated at 3000 ng/L, (reference range <125 ng/L)^3^ although he remained asymptomatic and performed well on the 6-minute walk test, achieving a distance of 500 m without limitation. A 12-lead electrocardiogram (ECG) showed sinus rhythm with pathological Q-waves mimicking prior anteroseptal infarction, and low QRS voltages (*Figure 1A*). Transthoracic echocardiography (TTE) revealed marked left ventricular (LV) hypertrophy, with an IVS thickness of 20 mm and relative wall thickness (RWT) of 0.57 (*Figure 1B*). LV ejection fraction (EF) was moderately reduced (41%). Doppler findings were consistent with elevated filling pressures (E/A ratio 2.8 and E/e′ ratio 25). Discordance between ECG voltage and echocardiographic findings prompted further investigation for cardiac amyloidosis. The patient was initiated on guideline-directed heart failure therapy, including Candesartan, Spironolactone, and Dapagliflozin. At the time, he was euvolemic, did not need diuretics, reported no overt cardiac symptoms, and reported normal exercise tolerance.

**Figure 2:**
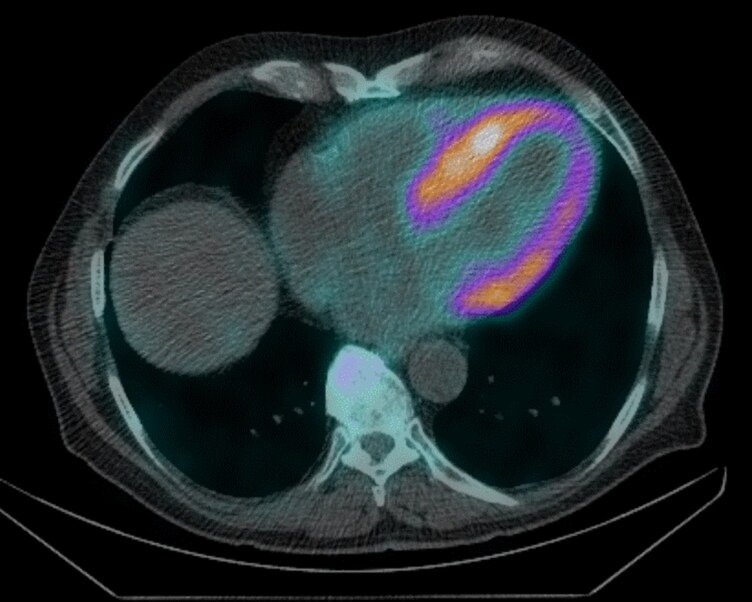
DPD scintigraphy with single-photon emission computed tomography (SPECT/CT) imaging demonstrating tracer accumulation predominantly in the basal LV wall.

Advanced echocardiography was performed in conjunction with DPD Scintigraphy. Speckle-tracking echocardiography (STE) showed impaired LV global longitudinal strain (GLS –10%) with apical sparing (RELAPS 2.8) and reduced LV myocardial work index (GWI) 956 mmHg% (*Figure 1C* and *D*). LA reservoir strain was impaired (16%). RV function was similarly compromised (RV free wall strain −16%, RV GWI 350 mmHg% (*Figure 1E* and *F*). DPD scintigraphy demonstrated grade 3 cardiac uptake on the Perugini scale, with predominant basal LV distribution, (*Figure 2*). Serum and urine immunofixation electrophoresis, together with a normal free light chain ratio, excluded AL amyloidosis. TR gene sequencing revealed no abnormalities, consistent with a diagnosis of ATTRwt-CM. Tafamidis therapy was recommended; however, initiation was delayed by approximately six months due to logistical barriers. During this period, the patient’s symptoms progressed, necessitating the introduction of diuretics to manage oedema and dyspnoea.

**Figure 3:**
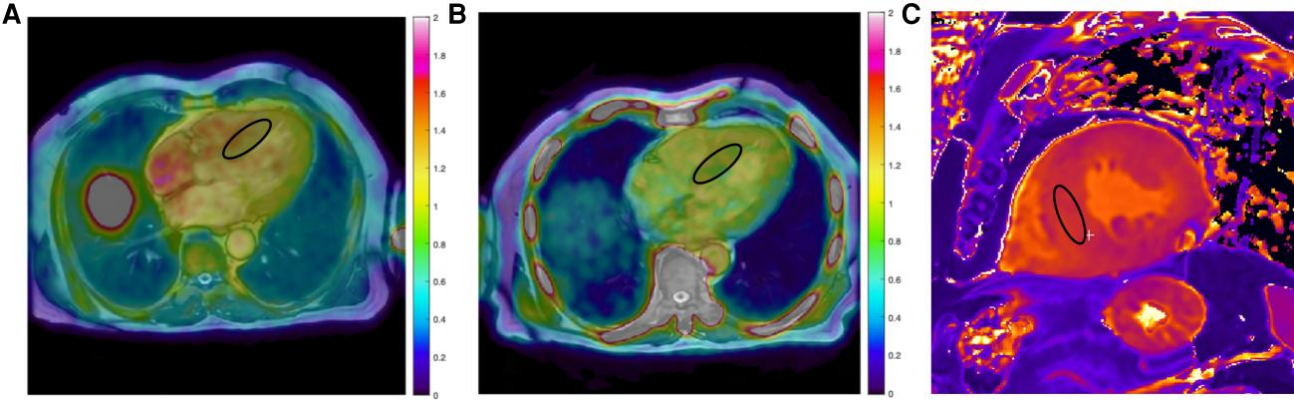
PET-MR images displaying demonstrating (*A* & *B*) myocardial uptake suggestive of amyloid infiltration and microcalcification, and (*C*) native T1 mapping by modified Look-locker inversion recovery (MOLLI) sequence, demonstrating diffusely increased native T1 values.

In September 2024, while still subjectively asymptomatic and as part of a research protocol, the patient underwent PET-MR and Invasive pressure–volume assessment. Cardiac PET imaging with ^18^F-flutemetamol demonstrated myocardial uptake suggestive of amyloid infiltration (SUVmean 1.24; TBR 1.0; *Figure 3A*). Cardiac MR showed elevated T1 and ECV values (1380 msec and 43%, respectively) (*Figure 3C*).

**Figure 4:**
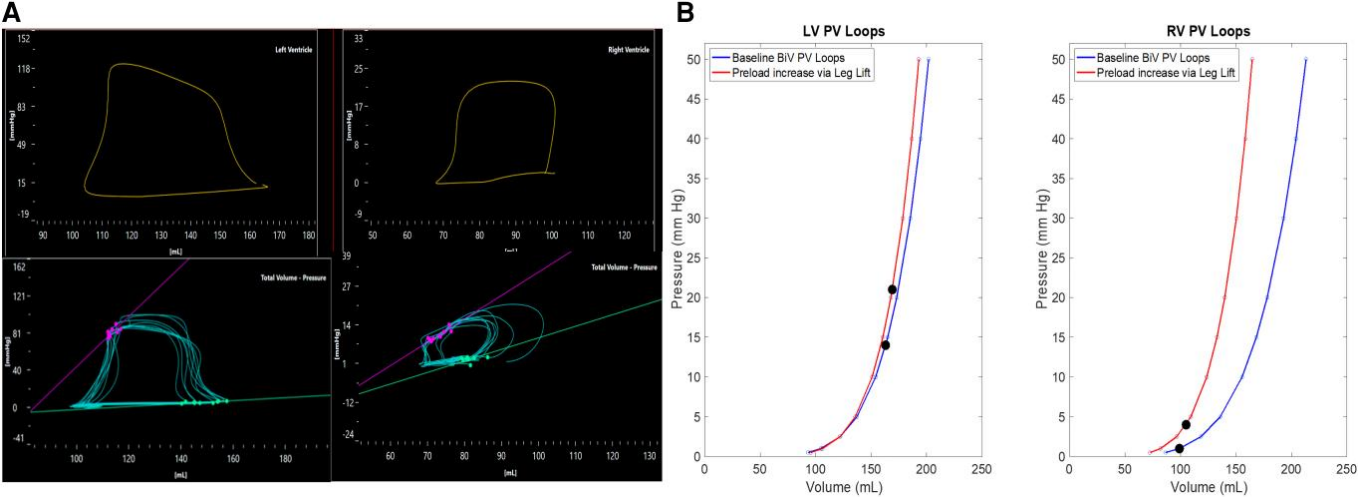
Invasive pressure–volume loop images displaying pressure–volume loops that confirmed impaired biventricular stiffness.

**Figure 5:**
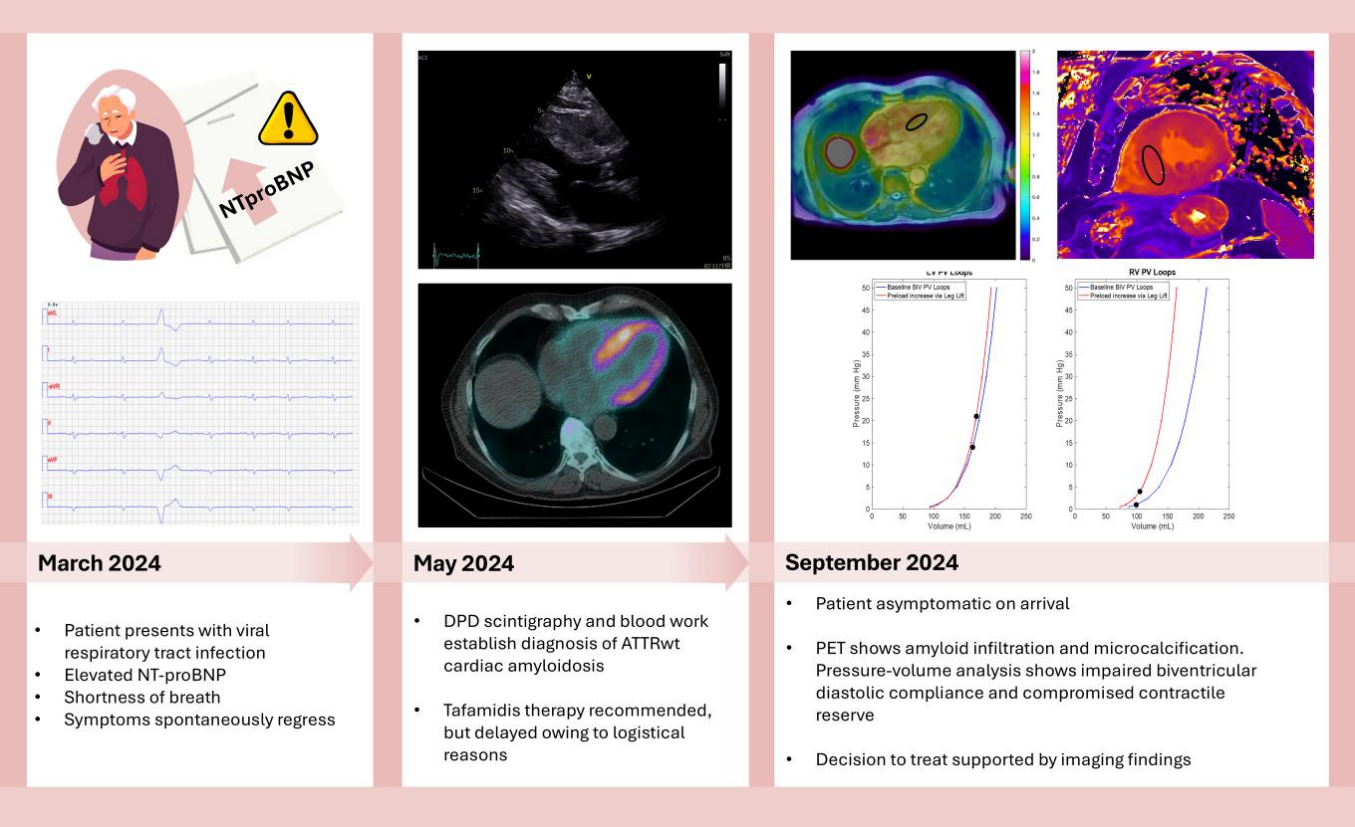
Timeline of diagnostic investigations during patient workup.

Invasive pressure–volume loop analysis confirmed impaired biventricular diastolic compliance, with prolonged relaxation times (LV Tau 40 ms, RV Tau 45 ms) and elevated Beta-stiffness constants (β: LV 5.9  ml^−1^, RV 5.1 mL^−1^; preload recruitable stiffness: LV 6.5 mmHg/mL, RV 5.6 mmHg/mL) (*Figure 4*). End-diastolic pressure–volume relationship (EDPVR) was normal at rest (LV 0.091 mmHg/mL, RV 0.044 mmHg/mL). Systolic function was preserved at rest, but contractile reserve was blunted during passive leg raising, as indicated by reduced RV end-systolic pressure–volume relationships (ESPVR: LV 1.0 mmHg/mL, RV 0.26 mmHg/mL) and modest preload recruitable stroke work slopes (PRSW: LV 56 mmHg, RV 46 mmHg). A timeline displaying key investigations during patient workup is presented in *Figure 5*.

## Discussion

This case report illustrates the powerful synergy of emerging diagnostic techniques in cardiac amyloidosis and the clinical challenge of managing patients with discordant functional status and objective disease severity. We present a comprehensive structural, functional, and metabolic profile of a patient with ATTRwt-CM, integrating PET-MR and invasive biventricular PV analysis. To our knowledge, this is the first reported case to integrate both advanced modalities in the evaluation of ATTRwt-CM.

**Added diagnostic value of PET-MR in ATTR-CM.** While bone scintigraphy remains the cornerstone for non-invasive diagnosis of ATTR-CM, PET-MR offers significant complementary value. Earlier studies have shown that PET-MR aids in the diagnosis of cardiac amyloidosis, identifies characteristic late gadolinium enhancement (LGE), and differentiation of ATTR and AL forms within a single, low-radiation scan.^4^ Studies indicate that certain TTR variants in ATTR may display low or absent ^99m^Tc-DPD uptake, likely reflecting differences in fibril types.^5^ While most ATTRwt cases display fragmented (Type A) fibrils, patients with the variant form may contain predominant full-length (Type B) fibrils, which are also associated with early onset of the disease. Emerging evidence suggests that PET imaging may be useful in this setting.^6^ Among available ^18^F-amyloid tracers, ^18^F-flutemetamol remains relatively less studied for the evaluation of cardiac amyloidosis. In a small study from our group evaluating patients with the V30M ATTR variant, we reported specificity and sensitivity values of 100% and 88%, respectively, using a septal SUV threshold of 1.46.^7^ PET imaging with ¹⁸F-flutemetamol revealed myocardial uptake consistent with amyloid deposition and possible microcalcification, suggesting that the high sensitivity of DPD scintigraphy in ATTR-CM may partly relate to binding of microcalcific components rather than amyloid fibrils.^8^

In ATTR-CM, characteristic LGE patterns progress from subendocardial to diffuse transmural as disease progresses and are accompanied by challenging blood–myocardium nulling on inversion recovery imaging. In recent years, ECV has emerged as a robust prognostic marker to assess treatment outcomes in ATTR-CM. The markedly elevated ECV in this case may reflect a poor prognosis.^9^ Integrating PET's metabolic quantification with CMR's tissue characterization creates a powerful tool for enhanced risk stratification and, potentially, for non-invasive monitoring of therapeutic response, particularly in equivocal diagnostic scenarios.

**Invasive Pressure–Volume Loop Analysis.** Invasive PV-loop analysis was first utilized to assess ventricular performance in 1895^10^ and provide highly quantitative, load-independent measures of myocardial mechanics. Validated non-invasive approaches have been employed in ATTR-CM to monitor treatment response^11^ and predict survival.^12^ In this case study, we highlight the role of invasive PV-loop analysis to unmask latent disease in a relatively asymptomatic patient. PV-loop analysis revealed significant impaired diastolic compliance and prolonged relaxation, quantified by elevated Beta-stiffness constants and prolonged Tau times. Importantly, the assessment unmasked blunted contractile reserve with preload augmentation, evidenced by reduced end-systolic pressure–volume relationships. This latent dysfunction substantiated that the functional reserve of the heart was compromised, justifying a disease stage beyond what symptoms or resting echocardiography suggested.

Currently, PV-loop analysis is not indicated for routine clinical practice due to its invasive nature, technical complexity, and limitation to highly specialized centres. However, its contribution in this case highlights a realistic, selective role: as a ‘gold standard’ staging tool in cases of significant clinical-imaging discordance, or to provide objective, quantifiable evidence of advanced functional impairment. This also supports justification for early initiation of expensive, disease-modifying therapies. In our case study, PV-loop data supported patient transition from a watchful waiting strategy to definitive, early therapeutic intervention.

**Imaging-Guided Decision Making in Complex Clinical Scenarios.** The presymptomatic nature of the disease posed a significant challenge to clinical decision-making, particularly in view of the substantial cost of disease-modifying therapy. In this case, multimodal imaging findings and biomarker profiles indicated more advanced myocardial involvement than suggested by the patient’s preserved functional status, as he remained asymptomatic while receiving MRA and SGLT2 inhibitor therapy. Based on the integrated imaging and haemodynamic evaluation, initiation of disease-modifying treatment was advised. Implementation, however, was delayed due to prescribing hesitancy at the regional level. Whether this delay contributed to the clinical deterioration observed in 2025 remains uncertain, as prior studies have shown that functional decline may occur despite treatment, particularly during the initial phase of Tafamidis therapy.^13^

This case underscores the emerging and complementary roles of PET-MR imaging and invasive haemodynamic assessment in the phenotyping of ATTRwt-CM. While PET imaging is particularly valuable in cases where DPD scintigraphy lacks sensitivity, it may also aid in distinguishing light-chain (AL) amyloidosis from ATTR-CM,^14^ and in detecting both amyloid infiltration and quantifying amyloid burden. Our case suggests that PET imaging with amyloid-targeting radiotracers may also identify associated microcalcification, potentially elucidating mechanisms behind DPD uptake in cardiac amyloidosis. Further, invasive haemodynamic assessment may offer enhanced sensitivity in detecting left ventricular stiffness and subclinical diastolic dysfunction beyond non-invasive modalities. These hypotheses warrant further investigation in larger, prospective studies to clarify diagnostic thresholds and clinical utility.

## Conclusion

This case demonstrates the utility of a multimodal approach where PET-MR informs on the structural and metabolic burden, and PV-loop analysis provides load-independent evidence of functional compromise. This integration allowed for precise phenotyping and justified the early therapeutic decision-making in a high-risk patient with ATTRwt-CM. Future prospective studies are warranted to define the specific clinical thresholds for PV-loop analysis and to clarify the role of PET-MR as a tool for monitoring disease progression and treatment efficacy.

## Patient perspective

I had been experiencing increasing fatigue and shortness of breath, which slowly affected my daily life. Although I felt relatively well at rest, the doctors recommended several advanced tests, including heart scans and a pressure assessment, to understand my condition better. At first, it was overwhelming, but the detailed imaging and evaluations gave me clarity about my heart health. Learning that I had wild-type transthyretin cardiac amyloidosis was surprising, yet it was reassuring to know that treatment was available. Starting Tafamidis early, even before severe symptoms appeared, gave me confidence that my condition could be managed proactively. Overall, I appreciate how the team used advanced diagnostics to guide personalized care, allowing me to maintain my independence and quality of life.

## Lead author biography


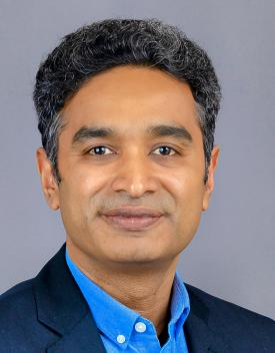


Ashwin Venkateshvaran, PhD, FASE, FESC is Associate Professor of Clinical Physiology and a Senior Staff Scientist at Umeå University, Sweden. His research focuses on advanced cardiac imaging - including echocardiography, cardiac MRI, PET and invasive hemodynamics - to improve early diagnosis, disease phenotyping and risk stratification in heart failure and infiltrative heart disease.

## Data Availability

All data underlying this article are available within the article itself. No additional datasets were generated or analysed for this case report.
